# Linking Seed Photosynthesis and Evolution of the Australian and Mediterranean Seagrass Genus Posidonia

**DOI:** 10.1371/journal.pone.0130015

**Published:** 2015-06-12

**Authors:** David Celdran, Javier Lloret, Jennifer Verduin, Mike van Keulen, Arnaldo Marín

**Affiliations:** 1 Unidad Académica de Sistemas Arrecifales (Puerto Morelos), Instituto de Ciencias del Mar y Limnología, Universidad Nacional Autónoma de México, Apto Postal 1152, CP: 77500, Cancún, Quintana Roo, Mexico; 2 Marine Biological Laboratory, The Ecosystems Center, Woods Hole, MA, United States of America; 3 School of Veterinary and Life Sciences, Environmental and Conservation Sciences, Murdoch University, Murdoch, WA, 6150, Australia; 4 Departamento de Ecología e Hidrología, Facultad de Biología, Universidad de Murcia, 30100, Murcia, Spain; Università della Calabria, ITALY

## Abstract

Recent findings have shown that photosynthesis in the skin of the seed of *Posidonia oceanica* enhances seedling growth. The seagrass genus *Posidonia *is found only in two distant parts of the world, the Mediterranean Sea and southern Australia. This fact led us to question whether the acquisition of this novel mechanism in the evolution of this seagrass was a pre-adaptation prior to geological isolation of the Mediterranean from Tethys Sea in the Eocene. Photosynthetic activity in seeds of Australian species of *Posidonia *is still unknown. This study shows oxygen production and respiration rates, and maximum PSII photochemical efficiency (Fv : Fm) in seeds of two Australian *Posidonia* species (*P*. *australis* and *P*. *sinuosa*), and compares these with previous results for *P*. *oceanica*. Results showed relatively high oxygen production and respiratory rates in all three species but with significant differences among them, suggesting the existence of an adaptive mechanism to compensate for the relatively high oxygen demands of the seeds. In all cases maximal photochemical efficiency of photosystem II rates reached similar values. The existence of photosynthetic activity in the seeds of all three species implicates that it was an ability probably acquired from a common ancestor during the Late Eocene, when this adaptive strategy could have helped *Posidonia* species to survive in nutrient-poor temperate seas. This study sheds new light on some aspects of the evolution of marine plants and represents an important contribution to global knowledge of the paleogeographic patterns of seagrass distribution.

## Introduction

The seagrass genus *Posidonia* has a unique fragmented distribution, with species found in Australia and the Mediterranean Sea, separated by about 17.000 km, but found nowhere else in the world. Western Australia contains some of the largest seagrass meadows on Earth, and has the highest diversity of *Posidonia* species (eight recognized species)[3, 6]. In the Mediterranean Sea, there is only one species, *Posidonia oceanica*.

**Fig 1:**
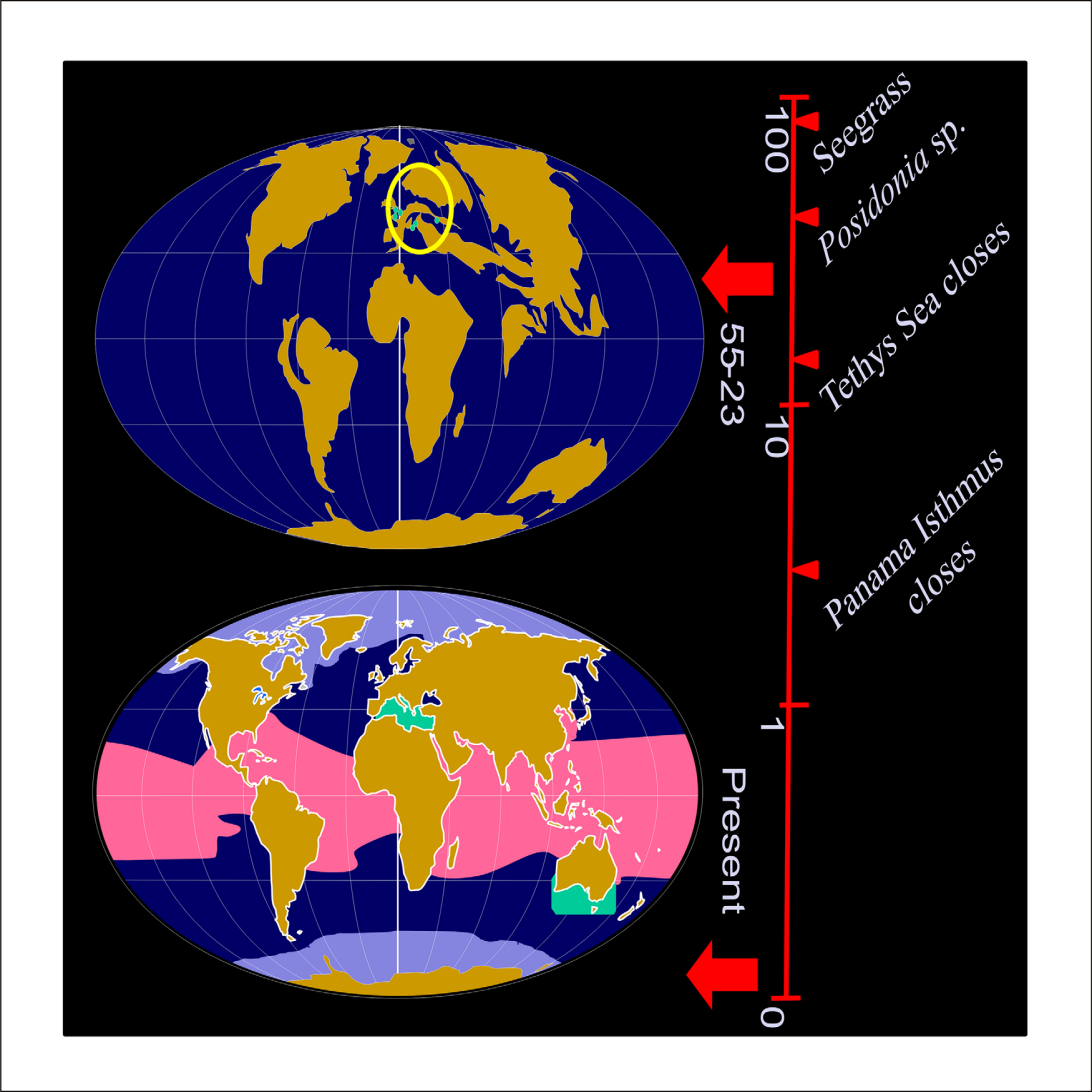
*Posidonia* Evolution. Posidonia evolution time line for the past 100 million years and current global distribution of Posidonia genus (in green color) in relation to mean ocean temperature. Climate differences as; Polar: purple color (< 4°C), Temperate: blue color (4°C–24°C), and Tropical: pink color (> 24°C). Green spots into the yellow circle point out the place with the first fossil Posidonia spp. records (Posidonia parisiensis), which are dated from middle Eocene to the top of the Oligocene (48.60000 to 23.03000 Ma).

*Posidonia* fossils found in Europe and recorded from the Cretaceous to the Miocene have remained almost unchanged over this long evolutionary history [5]. The nine current species of *Posidonia* of Australia and the Mediterranean Sea may have evolved from the Tethyan fossil species (e.g. *Posidonia cretacea*, *P*. *perforata*, and *P*. *parisiensis*) [13], Fig 1.

The reproductive cycle of *Posidonia* is sophisticated and complex, and reflects the evolutionary adaptation of a terrestrial plant to the sea and the environmental limiting factors for seedling colonization. *Posidonia* species release floating fruits that contain a single negatively buoyant seed that lacks dormancy. After fruit dehiscence, the green seed sinks to the bottom, where it rapidly develops primary roots and a leaf system. The green seed remains attached to the young plant for 1–2 years after germination. *P*. *oceanica* seeds display photosynthetic activity from the time of germination and throughout seedling development, which enhances seedling growth in leaves and roots. It was previously shown that 29.26% of leaf biomass and 42.31% of root biomass production was due to the seed´s photosynthetic activity in *P*. *oceanica* seedlings [4]. However, it is not known if the seeds of Australian species also have photosynthetic capacity. To find out, we studied and compared seed photosynthesis of Mediterranean (*P*. *oceanica*) and Australian (*P*. *australis* and *P*. *sinuosa*) species.

The existence of this capability in seeds of Australian species could indicate that this feature was developed before the closure of the Tethys Sea and could help us understand the ecology and evolution of *Posidonia* species, one of the longest and oldest organisms in the world’s oceans.

## Material and Methods

Fruits of *P*. *australis* and *P*. *sinuosa* were collected at Woodman Point, Perth, (SW. Australia), (32° 8.169' S 115° 44.521' E) on November 2011 while Mediterranean fruits of *P*. *oceanica* were collected at beaches of Mazarron, in Murcia (SE Spain), (37° 33´ N 1° 19´O) on May 2012, (n = 15 for each species). Due to *Posidonia* species release floating fruits, some of them arrive to the beach and can be collected directly from the seashore. Licence to take flora for scientific or other prescribed purposes” from the Western Australian Department of Environment and Conservation was: licence number SW013741. Likewise the permission of seed colection at Mediterranean beaches of Murcia was provided through the project "Restauración de praderas de Posidonia oceanica con plántulas; Influencia de la luz, nutrientes y herbivoría" (Number: TM2011-27377) by the Dirección General de Medio Ambiente of the Autonomous Comunity of Murcia (Spain).

Photosynthetic activity was assessed using an oxygen meter (HQ40d, Hach) to determinate the net production, gross production and respiration of seeds and an underwater pulse amplitude modulated fluorometer (Diving-PAM) to measure maximum PSII photochemical efficiency (Fv: Fm). Incubations for oxygen production were determined in dark and light and were carried out for 1 h. in a Versatile Environmental Test Chamber (Sanyo MLR-351) with controlled irradiance (300 μmol m^-2^ s^-1^) and temperature (19°C) which corresponds approximately to the seawater temperature where were collected (Australia and Spain). To compare seed sizes of the three species, 10 freshly collected seeds of each species were used to measure and compare dry weight.

We applied one-way ANOVA to (Fv: Fm), PN, PB, R and weight of seed data, to assess significant differences in seeds of the three species of Posidonia). If data was not parametric then a Kruskal-Wallis was applied. After one-way ANOVA and Kruskal-Wallis a Tukey HSD post-hoc test (α = 0.05) and a Dunns post-hoc test (α = 0.05) were run respectively.

## Results

The Mediterranean and Australian *Posidonia* seeds were similar in the green colour but they differ in shape and size. Seed dry weight of the Australian species was significantly less than the Mediterranean species (p<0.05), (Table 1), which suggests that the Mediterranean species invests more energy and stores more nutrients and carbohydrates in individual seeds than Australian species. There were significant differences in respiration, net oxygen production and gross oxygen production (p< 0.05) between the three species (Table 1). Post-hoc analysis showed that differences were due to a higher respiration and gross oxygen production in *P*. *oceanica* than in *P*. *australis* and *P*. *sinosa* respectively and higher net oxygen production in *P*. *australis* than *P*. *sinuosa* or *P*. *oceanica*.

**Table 1:** Fotobiologycal parameters and dry weight comparison of three *Posidonia* species.

	*P. australis*	*P. sinuosa*	*P. oceanica*	ANOVA
**R**	0.191 ± 0.032	0.217 ± 0.043	0.242 ± 0.051	p = 0.002
**N. P**	0.011 ± 0.035	-0.045 ± 0.058	-0.013 ± 0.007	p = 0.001
**G. P.**	0.202 ± 0.037	0.172 ± 0.049	0.229 ± 0.046	p < 0.001
**(Fv: Fm)**	0.647 ± 0.063	0.635 ± 0.071	0.673 ± 0.040	p = 0.270
**D. W.**	0.080 ± 0.019	0.061 ± 0.011	0.191 ± 0.060	p < 0.001

(R) Respiration, (N.P.) Net Production, (G.P.) Gross Production, (D.W.) Dry Weight and (Fv: Fm) maximum PSII photochemical efficiency *in P*. *australis*, *P*. *sinuosa* and *P*. *oceanica* seeds (mean ± s.d.). R, NP and GP were measured at 300 μmol m^-2^ s^-1^ and 19°C. Oxygen expressed as mg O2 gdw^−1^ h^−1^ and dry weight in grams. Results of ANOVAs (p values) comparing R, NP, GP, DW and (Fv: Fm) in the three species are included.

**Fig 2:**
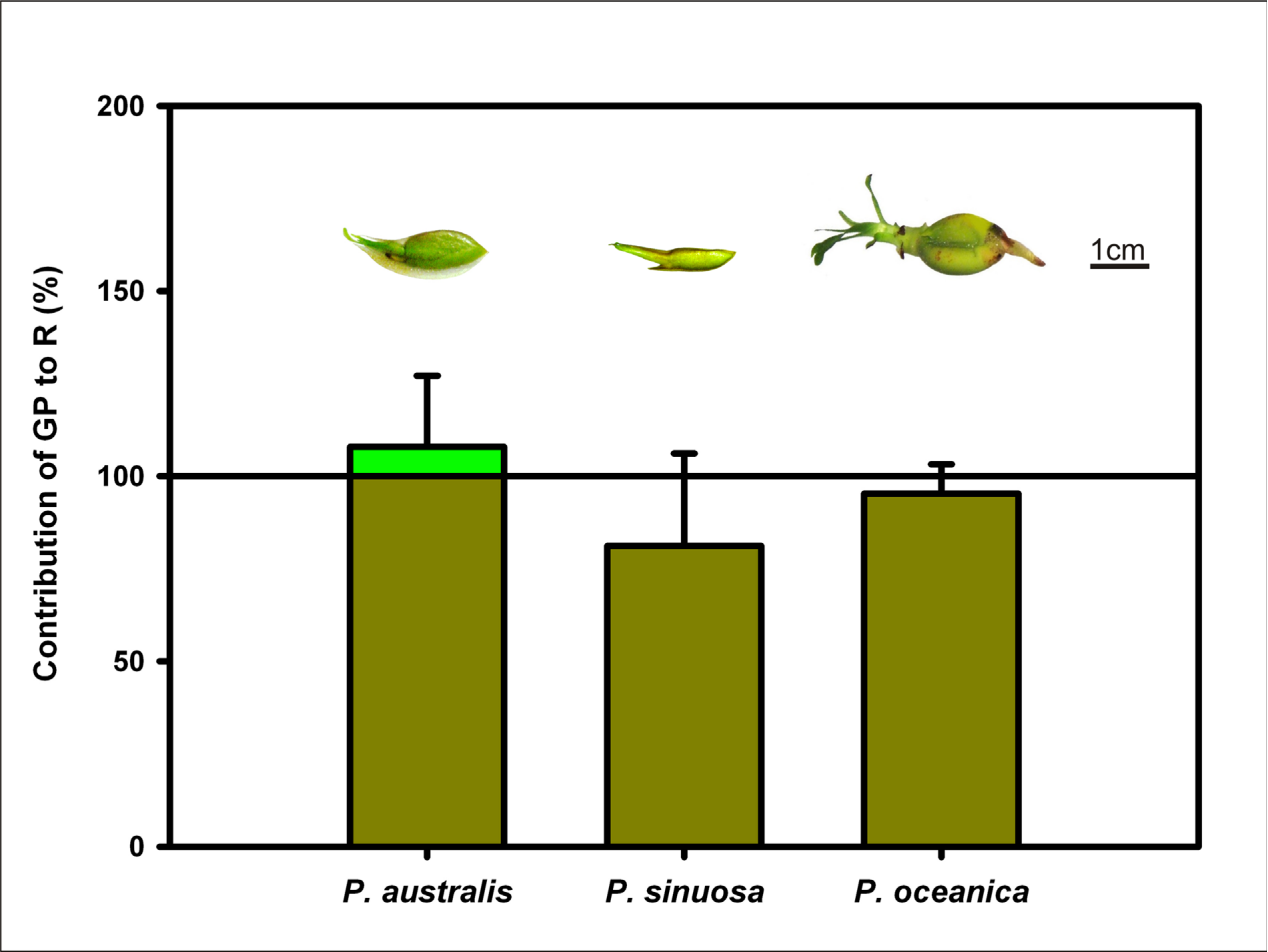
Contribution of Gross Production to Respiration. Contribution of Gross Production to Respiration (%) in seeds of *P*. *australis*, *P*. *sinuosa* and *P*. *oceanica*.

The contribution of seed photosynthesis to respiration was calculated from seed gross oxygen production and the respiratory demand. Despite *P*. *oceanica* having higher gross oxygen production than both Australian species, higher respiratory costs coming from the larger size of *P*. *oceanica* seeds means that *P*. *australis* seeds contribute greater oxygen production than the Mediterranean species (Fig 2). No differences were found between the three species in the maximum PSII photochemical efficiency (Fv: Fm), see Table 1.

## Discussion

Light and dark seed incubations demonstrated that both Australian and Mediterranean species display photosynthetic activity.

This photosynthetic activity seems to be a compensatory mechanism for the seed’s oxygen demands, since respiration was effectively balanced by photosynthesis in *P*. *sinuosa* and *P*. *oceanica* or even exceeded in the case of *P*. *australis* (Fig 2).

Photosynthesis during seed embryogenesis could be a mechanism for avoiding hypoxia, as observed in developing legumes [10, 15]. When the internal O_2_ level is low enough to limit respiration (i.e., hypoxia), raising the supply of O_2_ through seed photosynthesis could in principle alleviate this stress, by promoting the supply of respiratory energy [14]. Likewise seed photosynthesis in the *Posidonia* species studied could be a mechanism to alleviate the high respiration demand. The larger seed of *P*. *oceanica* increased respiration demand with respect to the two Australian species, and forced to optimize seed photosynthesis. Although *P*. *australis* has higher rates of oxygen production.

The no differences between the three species in maximum PSII photochemical efficiency (Fv: Fm) corroborates the fact that, despite millions of years of isolation, and despite they are not genetically close [8] and geographically separated by thousands of kilometres Mediterranean and Australian seeds of *Posidonia* species still display similar photosynthetic performance.

Photosynthetic activity occurs in seeds of different species of *Posidonia* from both geographical areas and this suggests that photosynthetic capacity was an adaptive mechanism acquired during the Late Eocene (37.2 million to 33.9 million years ago) from a common ancestor. Our data supports the hypothesis that this mechanism appeared when ancestral *Posidonia* successfully colonized nutrient-poor temperate habitats in the Tethys Sea. Photosynthesis in modern day seeds represent a mechanism to satisfy the high demand of oxygen required to metabolize the large reserves contained in the seed. This hypothesis would agree with the paleo-oceanographic history and the enclosure of Tethys Sea during the Miocene (20 to 10 Myr BP) that left the Mediterranean and Australian species isolated [11, 9]. The separation of the continents resulted in the isolation of the Mediterranean Sea in an enclosed basin connected to the Atlantic Ocean by the narrow Strait of Gibraltar, creating a poor nutrient habitat. *P*. *oceanica*, being a temperate species, may have been less affected by the closure of the Tethys Sea than were tropical species. However, other studies of phylogeny based on a nuclear marker (rRNA-ITS) revealed a more ancient split between the Mediterranean and Australian taxa (>60 Myr BP). Based on this evidence, the Australian *Posidonia* species are probably the survival descendants after the separation of Australia from Antarctica during the Eocene (55.8 to 33.9 Myr BP) [7, 16], followed by a more recent divergence of the two recognized Australian complexes around 12 Myr ago [1]. In the northern hemisphere only *P*. *oceanica* survived the enclosure and partial desiccation of the Mediterranean Sea during the Messinian salinity crisis [2], in putative Mediterranean refuge areas. There is a limited supply of nutrients to the surface waters of the Mediterranean Sea, both from its lower layers and from external sources (the Atlantic inflow, river discharges, atmospheric input) but the principal reason for this lack of nutrients is related to the Mediterranean’s hydrology and circulation as a concentration basin [12]. Only one species, *P*. *oceanica*, could adapt to this lack of nutrients, by increasing the nutritional reservoir of its seeds. This may be explained by the significant greater size and weight of the seed of *P*. *oceanica* than the Australian species.

On the other hand, Australian *Posidonia* species evolved along a broad latitudinal gradient of temperature. This allowed species to move gradually with the latitudinal advance and retreat of cooler conditions, which has favoured greater species diversity. Adaptation to the higher diversity of environmental conditions in Australian waters could explain the variability in seed size, nutrient content and photosynthetic activity of *P*. *australis* and *P*. *sinuosa*.

## Conclusions

In summary, the evidence of photosynthetic activity in mature and germinated seeds of *P*. *australis*, *P*. *sinuosa* and *P*. *oceanica* suggest that this photosynthetic activity is inherited from a common ancestor. The relatively large size of *P*. *oceanica* seeds compared to the two Australian species suggests that in the Mediterranean Sea, where a progressive but drastic basin closure occurred, *P*. *oceanica* maximized a strategy developed by these three species to survive in extreme oligotrophic conditions.

These findings allow us to redefine the evolutionary origins of this seagrass and provide an important contribution to global knowledge about the paleobiogeographic patterns and evolution of the species.
